# The Association between Dietary Vitamin A and Carotenes and the Risk of Primary Liver Cancer: A Case–Control Study

**DOI:** 10.3390/nu8100624

**Published:** 2016-10-11

**Authors:** Qiu-Ye Lan, Yao-Jun Zhang, Gong-Cheng Liao, Rui-Fen Zhou, Zhong-Guo Zhou, Yu-Ming Chen, Hui-Lian Zhu

**Affiliations:** 1 Department of Nutrition, School of Public Health, Sun Yat-sen University, Guangzhou 510080, Guangdong, China; bolivia.lan@gmail.com (Q.-Y.L.); liaogch3@mail2.sysu.edu.cn (G.-C.L.); ruifenzhou.sysu@gmail.com (R.-F.Z.); chenyum@mail.sysu.edu.cn (Y.-M.C.); 2 Department of Hepatobiliary Surgery, Sun Yat-sen University Cancer Center, Guangzhou 510080, Guangdong, China; zhangyuj@sysucc.org.cn (Y.-J.Z.); zhouzhg@sysucc.org.cn (Z.-G.Z.)

**Keywords:** vitamin A, carotenes, primary liver cancer

## Abstract

Dietary intake of vitamin A (VA) and carotenes has shown beneficial effects for decreasing the risk of some types of cancer, but findings on the risk of primary liver cancer (PLC) are inconsistent. This case–control study explored the associations between the dietary intake of VA and carotenes and the risk of PLC. We recruited 644 incident PLC patients (diagnosed within one month of each other) and 644 age- and gender-matched controls in Guangzhou, China. A food frequency questionnaire was used to assess habitual dietary intake. Logistic regression analyses found that higher intakes of VA and carotenes were independently associated with decreased PLC risk (all *P*_-trend_ < 0.001). The multivariable-adjusted ORs (95% CI) of PLC for the highest (vs. lowest) quartile were 0.34 (0.24–0.48) for vitamin A and 0.35 (0.25–0.49) for carotenes. The associations were not significantly modified by smoking, alcohol, or tea drinking (*P*_-interactions_: 0.062–0.912). Dose–response analysis showed a U-shaped VA–PLC relationship, with sharply decreased risks at the intakes of about 1000 μg retinol equivalent (RE)/day, and then slowly went down toward the flat-bottomed risks with the lowest risk at 2300 μg RE/day. Our findings suggest that greater intake of retinol, carotenes, and total VA may decrease PLC risk among the Chinese population at an intake of 1000 μg RE/day or greater from food sources.

## 1. Introduction

Primary liver cancer (PLC) is one of the most highly malignant tumors worldwide and is usually diagnosed at late stages with poor prognosis [1]. Approximately 50% of the total number of cases and deaths are estimated to occur in China [2]. Although chronic viral hepatitis (HBV and HCV) infection may account for the majority of PLC cases, an increasing number of studies has shown that some dietary factors may also be related to the development of PLC [3,4,5].

Vitamin A (VA) mainly comes from animal sources (retinol), and its precursor (carotenes) mainly from vegetables; it has substantial antioxidant effects and can increase the activity of detoxifying enzymes that combat the damage of reactive oxygen species [6] that may lead to oxidative DNA damage, followed by the initiation, promotion, and progression of carcinogenesis [7]. A study by Ramirez-Tortosa et al. found that the consumption of antioxidant micronutrients-rich foods may decrease DNA damage and increase antioxidant capacity [8]. However, inconsistent results were observed from human studies for the associations of vitamin A/retinol or its precursor with cancer risk. 

A few epidemiological studies have found favorable associations between the values of VA in diet or blood and the risk of various cancers. In a Shanghai cohort study [9], higher prediagnostic serum levels of retinol were related to a decreased risk of hepatocellular carcinoma (HCC). Similar favorable associations were observed in a large prospective study (the Alpha-Tocopherol, Beta-Carotene (ATBC) Cancer Prevention Study [10]) and in some case–control studies [11,12,13,14] between liver cancer beta-carotene and retinol levels in blood [10,11,12,13,14] and dietary sources [11]. However, null or even opposite results were observed in some studies. No significant associations with liver cancer were noted in two Chinese cohorts for dietary vitamin A [15] or for the supplementation of beta-carotene (20 mg/day) in the ATBC study [16]. Moreover, adverse effects of beta-carotene supplementation on other cancer risks were found in the ATBC Study [17], the Beta-Carotene and Retinol Efficacy Trial (CARET) [18], and were observed for other antioxidant nutrients (e.g., VE) in the Selenium and Vitamin E Cancer Prevention Trial (SELECT) [19]. Some experimental studies also indicated that antioxidant supplementation could increase the risk of cancer development and the progression of tumor cells in animal models [20,21]. Therefore, the associations of the levels of vitamin A and its precursor on the risk of cancer remain speculative. 

To address this issue, the present study examined the association between the dietary intake of retinol, carotenes, and their total retinol equivalent with PLC risk in a Chinese population. The generalized propensity score (GPS) approach was also used to estimate the dose–response effects of the probability of PLC and dietary intake of vitamin A for each level. 

## 2. Materials and Methods 

### 2.1. Study Population

This case–control study was conducted from September 2013 to January 2016 in Guangdong province, China. All newly-diagnosed PLC cases aged 18–80 years were recruited from Sun Yat-sen University Cancer Center. PLC patients were diagnosed within one month according to the National Comprehensive Cancer Network (NCCN) Clinical Practice Guidelines in Oncology: Hepatobiliary Cancers [22], and had not received any treatment before the recruitment. Case patients were excluded if they had the following conditions: (1) a history of other cancers; (2) confirmed type-2 diabetes; (3) significant changes in dietary habits or routine activities within the previous 5 years; and (4) incomplete dietary assessment or an implausible total daily energy intake (<700 or >4200 kcal per day for males, <500 or >3500 kcal per day for females). The control subjects were recruited from local communities with the same inclusion and exclusion criteria (except for liver cancer) in urban Guangzhou in the same time period. Finally, a total of 644 cases and 644 controls were considered in the present analysis. The Ethics Committee of the School of Public Health at Sun Yat-sen University approved the study protocol [EC_SPH_SYSU NO.17 (2012)]. Written informed consent was obtained from all participants at initial enrollment.

### 2.2. Data Collection

A personal interview was conducted by trained research interviewers using a structured questionnaire to collect the following information: socio-demographic characteristics (e.g., age, gender, education level, occupation, household income); lifestyle habits (e.g., smoking, alcohol drinking, tea drinking); habitual dietary intake and physical activities in the year prior to interview; and relevant diseases and medications. Individuals who drank tea at least twice weekly were considered as tea drinkers. Smokers or alcohol drinkers were defined as participants who smoked at least one cigarette per day or drank alcohol at least once a week continuously for at least six months. A 19-item questionnaire—including questions on daily, occupational, and leisure time activities—was used to evaluate the participants’ daily physical activity, reported as metabolic equivalent hours per day (MET h/day). Education level was divided into secondary school or below and high school or above. Household income was grouped into three levels (≤2000, 2001–6000, and >6000 Yuan/month/person). Anthropometric measurements included body height and weight and circumferences at the waist, hip, and neck. Body mass index (BMI, kg/m^2^) and waist-to-hip ratio (WHR) were then calculated.

### 2.3. Dietary Assessment

Dietary intake was obtained from a 79-item food frequency questionnaire (FFQ) by a face-to-face interview. The information covers the intake frequency (never, per year, per month, per week, or per day) and portion size of each item one year prior to diagnosis for PLC patients or one year prior to the time of interview for controls. Common food pictures in usual portion sizes were available to help the participants quantify the amount. Food intakes were converted into a daily intake of grams per day. Daily dietary nutrient intakes, including total energy, retinol, carotenes, total vitamin A (in retinol equivalent, RE), and other nutrients were calculated based on the China Food Composition Table 2004 [23]. Dietary intake of total vitamin A was calculated as total retinol equivalents by “retinol (in μg) + β-carotene (in μg)/12”. The validity and reproducibility of the FFQ were confirmed by three-day dietary records at intervals of two months during a 12-month period and two FFQs administered one year apart among 61 female subjects recruited from the same region. The energy-adjusted correlation coefficients between two FFQs of vitamin A, carotenes, and retinol were 0.57, 0.55, and 0.56, respectively. The energy-adjusted correlation coefficients comparing the second FFQ and 18 d dietary records were 0.32 for vitamin A, 0.32 for β-carotene, and 0.31 for retinol [24].

### 2.4. Generalized Propensity Score 

The generalized propensity score (GPS) approach is an alternative to regression for estimation of the dose–response function of continuous values or multivalued treatments, for evaluation of the treatment level received, and for observation of the covariates [25]. The key feature of the GPS is that its balancing property is similar to the propensity score of binary treatments and to answering complex questions in non-experimental settings [26]. Polychotomizing a continuous variable in regression models often leads to a loss of information and raises important problems when interpreting the magnitude of the association, which mostly depends on the cut-point. Thus, in our present study, we used the continuous variable, as well as the categorical variable of dietary intake, to test the relationship between VA and PLC by using GPS, which might balance the bias of dietary choice among participants and other selection biases confounding the outcome, and might ensure the reliability of the results.

### 2.5. Statistical Analysis 

All analyses were performed for men and women combined. Dietary intake of carotenes and retinol was adjusted for total calories using the residual method [27]. *t*-test, chi-squared test, and Wilcoxon rank-sum test were used to test differences in socio-demographic and nutrient intakes between the case and control subjects as appropriate. Logistic regression was used to estimate odds ratios (OR) and the corresponding 95% confidence intervals. The consumption of nutrients or food was divided into quartiles (Q1–Q4) according to the corresponding distribution among controls by gender, and odds ratios of PLC were calculated by quartiles of dietary intake, with the lowest consumption category as the reference. We adjusted for sex, age, BMI, education level (secondary school or below and high school or above), income level (≤2000, 2001–6000, >6000 Yuan/month/person), currently smoking (yes or no), currently drinking alcohol (yes or no), currently drinking tea (yes or no), and physical activity (MET h/day). Linear trends across increasing quartiles were tested by assigning quartiles as continuous variables in the regression analyses. *P* were based on two-sided tests and considered significant at <0.05.

To test the robustness of models, we also conducted two sensitivity analyses. First, we conducted stratified analyses to determine whether the above-mentioned associations were modified by lifestyle behaviors (smoking, alcohol use, and tea drinking). Secondly, we tested the relationship between intake of VA and PLC by dose–response functions using the GPS method [25], which is defined as the conditional probability of a response variable (i.e., PLC) at each particular level of the exposure (or treatment) (i.e., vitamin A) given the pre-“exposed” variables (covariates), such as sex, age, BMI, education and income level, smoking, alcohol drinking, tea drinking, physical activity, and energy intake. GPS was analyzed using Stata 12.0, and other data were analyzed using R 3.2.3.

## 3. Results

### 3.1. Participant Characteristics 

Selected characteristics of cases and controls are summarized in Table 1. There were 644 case–control pairs, including 85 female pairs and 559 male pairs. The median age was 54.3 years in the PLC cases and 54.4 years in the controls. PLC patients had lower values of BMI, WHR, physical activity, and a lower proportion of tea drinkers, but higher proportions of smokers and alcohol users compared with the controls (all *p* < 0.05). 

### 3.2. Dietary Intakes

PLC patients consumed less energy (1523 vs. 1635, kcal/day, *p* < 0.05), and lower energy-adjusted intakes of VA, carotenes, and retinol compared to controls (all *p* < 0.001). The top five food sources of retinol were animal offal (54.0%), eggs (20.3%), dairy products (5.7%), lean hogs (3.9%), and freshwater fish (3.6%). Leafy green vegetables contributed to more than half of carotene intake (73.1%), followed by carrots, melon, and fruit vegetables, tomatoes, and citrus fruits (Table 2). The proportions of the top five food sources of retinol and carotenes among case subjects were largely similar to those of the control subjects (Table 2).

### 3.3. Associations between Dietary Intakes of Vitamin A, Carotenes, and Retinol and PLC Risk

Table 3 presents the ORs and 95% CIs for PLC risk according to quartiles of VA, carotenes, and retinol consumption. Univariate unconditional logistic regression analyses reported a dose-dependent inverse correlation between PLC risk and all three nutrients (*P*_-trend_ < 0.001). In multivariable analysis, the ORs (95% CI) of PLC for the highest (vs. lowest) quartiles of intakes were 0.34 (95% CI: 0.24–0.48; *P*_-trend_ < 0.001) for VA, 0.35 (95% CI: 0.25–0.49; *P*_-trend_ < 0.001) for carotenes, and 0.37 (95% CI: 0.27–0.52; *P*_-trend_ < 0.001) for retinol.

We further analyzed the association of major food sources (those that accounted for more than 10% of the total sources) of retinol and carotenes with PLC risk (supplementary materials Table S1). Leafy green vegetables, carrots, eggs, and animal offal were inversely associated with the risk of PLC (all *P*_-trend_ < 0.05) in multivariable analysis. Egg intake was also found to be inversely associated with PLC risk, but no statistical significance was observed after adjusting for other factors.

### 3.4. Stratified Analyses

The favorable associations between dietary values of VA, carotenes, and retinol and PLC risk remained significant in all subgroups stratified by smoking, alcohol use, and tea drinking (all *P*_-trend_ < 0.05). The associations were not significantly modified by the status of smoking, alcohol, or tea drinking (all *P*_-interaction_ > 0.05) (Table 4).

### 3.5. Generalized Propensity Score (GPS)

**Figure 1:**
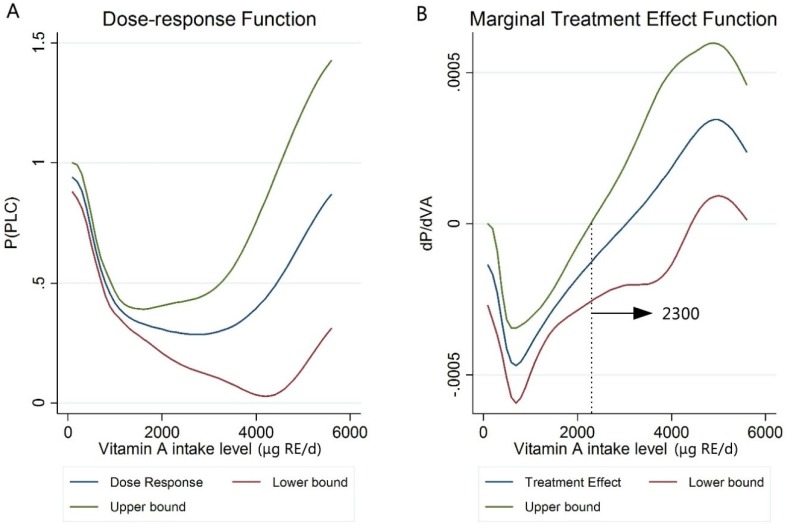
Dose–response function and marginal treatment effect function (dP/dVA) of expected probability of PLC and vitamin A intake (in μg retinol equivalent (RE)/day, 1 μg RE = 3.3 IU) using the generalized propensity score (GPS) approach, point estimate, and 95% CI. (**A**) The relationship between VA intake and the expected probability of PLC; (**B**) marginal treatment effect function of VA intake and the expected probability of PLC association. Lower and upper bound: 95% confidence interval; P (PLC) = the expected probability of PLC; dP/dVA = the derivating dose–response function.

Furthermore, we estimated the dose–response functions for the probability of PLC according to the values of VA using the GPS approach. We observed a U-shaped relationship between VA and PLC risk, in which the PLC risk decreased sharply under intakes of around 1000 μg RE/day, and then slowly went down toward flat-bottomed risks under a range of 1500–3000 μg RE/day (Figure 1A). Marginal treatment effect function analysis (which can be interpreted as a derivate) showed that the point estimated of VA intake with the lowest risk of PLC is 2300 μg RE/day (Figure 1B). 

## 4. Discussion

In this case–control study with the largest number of PLC cases published, we found that higher dietary intakes of vitamin A, carotenes, and retinol were independently associated with a lower risk of PLC. Dose–response analysis showed a U-shaped VA–PLC relationship with sharply decreased risks under the intakes of around 1000 μg RE/day, and then slowly went down toward flat-bottomed risks at the intake of 2300 μg RE/day. Our findings suggest that moderate dietary intake of vitamin A (under 2300 μg RE/day) could be beneficial for the prevention of PLC in this population. An amount of 100–300 g/day of carotene-rich vegetables (e.g., carrot, broccoli, spinach, etc.) or about 700 g/day of carotene-rich fruit (orange, mango, papaya, etc.) may meet the need for the plateau intake of 1000 μg RE/day (supplementary materials Table S2).

Many epidemiological studies have examined associations between the values of VA in diet or blood and the risk of liver cancer or other cancers. Favorable associations were observed in some (but not all) studies. In a prospective Shanghai study among 213 HCC patients and 1087 matched control subjects from a cohort of 18,244 men [9], higher prediagnostic serum levels of retinol were associated with a lower HCC risk. Similar favorable associations were observed in a large prospective study (the ATBC study) in 208 PLC cases from 29,046 men with baseline serum retinol/beta-carotene data, followed up for 24-years [10]. A few retrospective case–control studies also showed beneficial associations between liver cancer and beta-carotene and retinol levels in blood [10,11,12,13,14] and for the relevant dietary markers [11]. However, null or even opposite results were observed in some studies. No significant associations with liver cancer were noted in two Chinese cohorts for dietary vitamin A in 118 female PLC cases from 74,941 women, and 149 male cases from 61,491 men in Shanghai [15], and for the supplementation of beta-carotene (20 mg/day) and α-tocopherol (50 mg/day) for 5–8 years and followed up for over 24 years in the ATBC study in a randomized trial of 29,105 Finnish male smokers [16]. Moreover, increased risk of lung cancer was observed in the participants with beta-carotene supplementation from the ATBC Study [17] and CARET study (30 mg beta-carotene and 25,000 IU of retinol for five years) [18]. 

The results of our study were consistent with the majority of the observational studies, supporting that the dietary intakes of vitamin A and carotenes were inversely associated with PLC risk in humans. The possible reasons for the between-study heterogeneity may be due to the variations in study design, exposure makers, exposure dosage, and covariate adjustments. Favorable associations tended to be more frequently observed in case–control [11,12,13,14] (vs. cohort [15]) studies, in observational [10,11,12,13,14] (vs. interventional [16,17,18]) studies, in those using circulating [10,11,12,13,14] (vs. dietary intake [11,15]), and with low to moderate [15,28] (vs. high [17,18]) exposure or interventional dosages. Typically, blood measurements of retinol or beta-carotene had much higher precision in the determination of the internal doses than their dietary assessments, due to much smaller random error. Moreover, circulating markers may also eliminate the greater variability in the bioavailability of dietary/supplemental retinol or beta-carotene [29]. Therefore, the studies using blood markers had larger power to detect the statistical associations due to lower between-individual variations in the exposure determination. Regarding the study design, observational studies usually had moderate exposure dosages of vitamin A/beta-carotene, compared to interventional studies that consumed at least five-times the beta-carotene of those in habitual diets. Previous studies suggested that extremely low or high levels of vitamin A might promote tumor development and metastasis of transplantable hepatocellular carcinomas in rats [30]. Our findings also suggested a U-shaped VA–PLC relationship, suggesting that moderate rather than high levels of vitamin A might be beneficial for cancer prevention. The adverse effects of beta-carotene/VA supplementation on cancer risk might be due to smoking, although we could not exclude the possibility of a causal relationship. A meta-analysis showed that high-dose beta-carotene supplementation seemed to increase the risk of lung cancer among current smokers (OR 1.24, 95% CI 1.10–1.39) but not in former smokers (OR 1.10, 95% CI 0.84–1.45) [31], while the beneficial association of dietary intake of carotenoids with lung cancer was mainly observed among never-smokers [28]. Another reason for the differences between observational and interventional studies might be related to residual confounders (e.g., beneficial dietary components and heathy behaviors or other lifestyle factors) that associated with vitamin A/beta-carotene, but could not be excluded in observational studies. 

The potential mechanisms for the beneficial effects of vitamin A on PLC may involve antioxidant effects. Oxidative damage has been proven to play a key role in human cancer development [32]. Most of the PLC patients also had chronic infection with hepatitis B or C virus [33], which could cause significant impairments in antioxidant factors, generating reactive oxygen species (ROS) and reactive nitrogen species (RNS) [32,34,35]. Moreover, increasing levels of 8-hydroxydeoxyguanosine (8-OhdG)—an index of oxidative DNA damage—in DNA from livers with chronic hepatitis were also found in early animal studies [36,37]. Vitamin A and its retinoid derivate are important antioxidants. Generally, vitamin A was stored in the liver, and hepatic stellate cells (HSC) were the major sites. Indeed, it was confirmed that activated hepatic stellate cells could provoke a range of liver injuries associated with hepatic fibrogenesis, cirrhosis [38], and hepatocellular carcinoma [39]. Moreover, stored intracellular vitamin A loss occurs following the activation of HSCs [40]. Several studies have also demonstrated the vitamin A malnutrition status in patients with chronic liver disease [6,41,42]. However, there have been some studies reporting that antioxidant supplementations would accelerate the proliferation and metastasis of some particular tumor cells in mice models [20,21]; the potential mechanism of these studies was that antioxidants could alleviate oxidative stress, which not only harms normal cells but also limits tumor cell migration, invasion, and metastasis. These studies were performed on mice that had already induced cancer and were based on a relatively high intake of antioxidant supplementation. As mentioned above, trials in humans demonstrated that supplementation with beta-carotene and vitamin A could increase the risk of lung cancer [16,17], but increased vitamin A intake from food could have inverse effects on some cancer incidence [13,25]. These findings may suggest that moderate antioxidant intake is preferable, and that high intakes of antioxidants are unsafe. Therefore, it can be inferred that vitamin A may have an important role in PLC prevention and progression, which is consistent with our finding that a greater intake of vitamin A in a moderate range was significantly related to reduced PLC risk.

The strengths of this study include: (1) relatively large number of PLC cases in the VA–PLC association studies; (2) we included only newly-diagnosed PLC cases to prevent the possible incident–prevalent bias; and (3) the GPS method was used to estimate the dose–response function, in which we found that a moderately high intake of 1000–2300 μg RE/day would be helpful for PLC prevention. Compared with logistic regression, the GPS method can estimate the average risk of PLC at continuous levels of exposures of dietary intakes of VA, and the GPS method removes all bias associated with differences in the given pre-treatment variables (covariates) [43].

Several limitations of this study cannot be ignored. Firstly, PLC patients were all recruited from Sun Yat-sen University Cancer Center. However, the clinical characteristics of the PLC patients in this hospital were similar to patients in other areas of China [44,45], suggesting the potential representativeness of the PLC case patients in our study. Secondly, the controls were recruited in the communities in Guangzhou city. Part of the controls did not match the source of regions of PLC patients. We compared the VA–PLC associations between the case–control pairs with cases from Guangzhou and from other regions of Guangdong province and found non-significant between-region differences (*P*_-interaction_ = 0.299, 0.464, and 0.226 for VA, carotenes, and retinol). Thirdly, recall bias might be unavoidable in case–control studies, although we provided food photographs with portion sizes to help participants quantify intakes. Fourthly, our study used a FFQ to collect dietary intake information, and FFQs are known to contain a certain degree of measurement error, although the method has been validated in previous studies [46,47,48]. Fifthly, we could not infer the potential causal relationship due to the case–control study design, even though we recruited only new cases and excluded those cases and controls with significant changes in their dietary habits in the last five years to avoid the possibility of a reversed causal relationship between VA intake and PLC risk. Finally, we could not exclude the possibility that the favorable associations might be caused by the co-existing food components, healthy behavior, or other lifestyles associated with retinol/beta-carotene (residual confounding) in such an observational study. However, the results remained largely the same with or without the adjustments for a wide range of potential confounders (such as sex, age, BMI, education level, marital status, income level, smoking, alcohol drinking, tea drinking, physical activity, Table 3), and stratified by the status of smoking, alcohol drinking, and tea drinking (*P*_-interaction_: 0.062 to 0.912, Table 4) .

## 5. Conclusions

In conclusion, our study suggests that higher dietary consumption of retinol, carotenes, and total vitamin A (in RE) were independently and inversely associated with the risk of primary liver cancer risk. The intake of 1000 μg RE/day of vitamin A is approaching the ceiling effect on the prevention of PLC, although we got an intake of 2300 μg RE/day of total dietary vitamin A at the lowest PLC risk in this population. Further prospective (particularly interventional) studies are needed to confirm whether moderate doses of RE were beneficial for the prevention of liver cancer in humans.

## Figures and Tables

**Table 1:** Comparison of general characteristics between primary liver cancer cases and controls.

Characteristics	PLC (*n* = 644)	Controls (*n* = 644)	*p*
Age (years) ^1^	54.33 (10.34)	54.36 (10.23)	0.961 *
Gender (%)			
Male	559 (86.8)	559 (86.8)	1 *^#^*
Female	85 (13.2)	85 (13.2)	
BMI (kg/m^2^) ^1^	22.84 (3.33)	23.34 (3.16)	0.005 *
WHR ^1^	0.91 (0.07)	0.93 (0.06)	0.001 *
Physical activity (MET h/per day) ^1^	32.91 (13.29)	37.46 (9.61)	<0.001 *
Education level, *n* (%)			
Secondary school or below	384 (59.6)	399 (62.0)	0.424 *^#^*
High school or above	260 (40.4)	245 (38.0)	
Household income (Yuan/month), *n* (%)			
≤2000	230 (35.7)	254 (39.4)	0.094 ^+^
2001–6000	333 (51.7)	325 (50.5)	
>6000	81 (12.6)	65 (10.1)	
Smoking, *n* (%)	345 (53.6)	283 (43.9)	0.001 ^#^
Alcohol user, *n* (%)	208 (32.3)	125 (19.4)	<0.001 ^#^
Tea Drinker, *n* (%)	353 (54.8)	398 (61.8)	0.011 ^#^
Energy intake (kcal/day) ^2,4^	1523 (1224, 1865)	1635 (1255, 1938)	0.018 ^+^
VA (μg RE/day) ^3,4^	824 (577, 1150)	1024 (753, 1421)	<0.001 ^+^
Carotenes (μg/day) ^3,4^	3486 (2276, 5152)	4556 (3123, 6356)	<0.001 ^+^
Retinol (μg/day) ^3,4^	150 (87, 262)	191 (125, 308)	<0.001 ^+^

^1^ Continuous values are mean (standard deviation); ^2^ Energy intakes exclude cooking oil; ^3^ Energy-adjusted intakes; ^4^ Values are median (P25, P75); * *p*-values, two-sided *t*-test; *^#^ p*-values, two-sided chi-square test; ^+^ *p*-values, Wilcoxon rank-sum test; Abbreviation: MET = metabolic equivalent; PLC: primary liver cancer; VA: vitamin A; WHR = waist-to-hip ratio.

**Table 2:** Top five food sources of retinol and carotenes among case and control subjects.

Food Sources	Proportion (%)	
	Cases	Controls
Retinol		
Animal offal	52.8	54.0
Eggs	20.9	20.3
Dairy products	3.9	5.7
Lean hogs	4.8	3.9
Freshwater fish	3.7	3.6
Sub-total	86.1	87.5
Carotenes		
Leafy green vegetables	70.5	73.1
Carrots	15.7	13.3
Melon and fruit vegetables	4.3	3.9
Tomatoes	2.3	2.7
Citrus fruits	1.8	1.7
Sub-total	94.6	94.7

**Table 3:** Odds ratios (OR) and 95% confidence intervals (CI) of primary liver cancer according to quartiles of vitamin A, carotenes, and retinol intake.

	Amount	*n* (Cases/Controls)	Crude OR (95% CI)	Adjusted OR (95% CI) #
Quartile of VA (μg RE/day)				
Q1	≤753	276/161	1.00	1.00
Q2	753–1024	149/161	0.54 (0.40–0.73) ***	0.53 (0.39–0.73) ***
Q3	1024–1421	122/161	0.44 (0.33–0.60) ***	0.44 (0.32–0.60) ***
Q4	>1421	97/161	0.35 (0.26–0.48) ***	0.34 (0.24–0.48) ***
*P*_-trend_			<0.001	<0.001
Quartile of Carotenes (μg/day)				
Q1	≤3123	272/161	1.00	1.00
Q2	3123–4556	163/161	0.60 (0.45–0.80) **	0.56 (0.41–0.77) ***
Q3	4556–6356	110/161	0.40 (0.30–0.55) ***	0.41 (0.29–0.57) ***
Q4	>6356	99/161	0.36 (0.27–0.50) ***	0.35 (0.25–0.49) ***
*P*_-trend_			<0.001	<0.001
Quartile of Retinol (μg/day)				
Q1	≤125	266/161	1.00	1.00
Q2	125–191	146/161	0.55 (0.41–0.74) ***	0.49 (0.36–0.68) ***
Q3	191–308	108/161	0.41 (0.30–0.56) ***	0.34 (0.24–0.48) ***
Q4	>308	124/161	0.47 (0.34–0.63) ***	0.37 (0.27–0.52) ***
*P*_-trend_			<0.001	<0.001

** *p* < 0.01, *** *p* < 0.001; #: adjusted for sex, age, BMI, education level, income level, smoking, alcohol drinking, tea drinking, and physical activity.

**Table 4:** Associations between quartiles of vitamin A, carotenes, and retinol intake by controls and PLC risk according to smoking and alcohol use.

		Q1	Q2	Q3	Q4	*P_–_*_trend_
VA						
Smoking						
Yes	*n* (cases/controls)	149/70	85/72	63/70	48/71	
	OR (95% *CI*) ^1^	1.00	0.55 (0.35–0.86) *	0.37 (0.23–0.60) ***	0.32 (0.20–0.53) ***	<0.001
No	*n* (cases/controls)	121/90	72/91	55/90	51/90	
	OR (95% *CI*) ^1^	1.00	0.54 (0.34–0.85) **	0.42 (0.26–0.67) ***	0.37 (0.23–0.59) ***	<0.001
*P*_–interaction_						0.657
Alcohol use						
Yes	*n* (cases/controls)	98/31	61/32	29/31	20/31	
	OR (95% *CI*) ^1^	1.00	0.57 (0.30–1.1) ^+^	0.30 (0.15–0.60) **	0.22 (0.10–0.47) ***	<0.001
No	*n* (cases/controls)	178/129	100/131	83/130	75/129	
	OR (95% *CI*) ^1^	1.00	0.54 (0.38–0.78) **	0.46 (0.31–0.67) ***	0.39 (0.27–0.58) ***	<0.001
*P*_–interaction_						0.062
Tea drinking						
Yes	*n* (cases/controls)	144/100	86/99	70/99	53/100	
	OR (95% *CI*) ^1^	1.00	0.60 (0.39–0.92) *	0.52 (0.34–0.81) **	0.40 (0.25–0.63) ***	<0.001
No	*n* (cases/controls)	132/62	66/61	47/61	46/62	
	OR (95% *CI*) ^1^	1.00	0.41 (0.25–0.68) ***	0.33 (0.19–0.55) ***	0.34 (0.20–0.58) ***	<0.001
*P*_–interaction_						0.343
Carotenes						
Smoking						
Yes	*n* (cases/controls)	142/71	102/71	56/70	45/71	
	OR (95% *CI*) ^1^	1.00	0.64 (0.41–1.00) ^+^	0.36 (0.22–0.58) ***	0.31 (0.19–0.52) ***	<0.001
No	*n* (cases/controls)	122/90	69/90	54/91	54/90	
	OR (95% *CI*) ^1^	1.00	0.56 (0.35–0.88) *	0.43 (0.27–0.69) ***	0.40 (0.25–0.65) ***	<0.001
*P*_–interaction_						0.375
Alcohol use						
Yes	*n* (cases/controls)	91/31	68/32	30/31	19/31	
	OR (95% *CI*) ^1^	1.00	0.54 (0.28–1.04) ^+^	0.29 (0.14–0.59) **	0.18 (0.08–0.39) ***	<0.001
No	*n* (cases/controls)	176/129	105/131	76/129	79/130	
	OR (95% *CI*) ^1^	1.00	0.57 (0.39–0.81) **	0.43 (0.29–0.64) **	0.41 (0.28–0.61) ***	<0.001
*P*_–interaction_						0.069
Tea drinking						
Yes	*n* (cases/controls)	147/100	89/99	64/99	53/100	
	OR (95% *CI*) ^1^	1.00	0.57 (0.37–0.88) *	0.49 (0.31–0.76) **	0.38 (0.24–0.60) ***	<0.001
No	*n* (cases/controls)	129/62	70/61	46/61	46/62	
	OR (95% *CI*) ^1^	1.00	0.46 (0.28–0.75) **	0.34 (0.20–0.57) ***	0.34 (0.20–0.57) ***	<0.001
*P*_–interaction_						0.070
Retinol						
Smoking						
Yes	*n* (cases/controls)	125/70	79/71	70/71	71/71	
	OR (95% *CI*) ^1^	1.00	0.56 (0.35–0.90) *	0.42 (0.25–0.69) **	0.43 (0.27–0.70) **	<0.001
No	*n* (cases/controls)	142/90	66/91	38/90	53/90	
	OR (95% *CI*) ^1^	1.00	0.41 (0.26–0.64) ***	0.24 (0.15–0.41) ***	0.30 (0.19–0.49) ***	<0.001
*P*_–interaction_						0.484
Alcohol use						
Yes	*n* (cases/controls)	83/31	58/32	31/31	36/31	
	OR (95% *CI*) ^1^	1.00	0.61 (0.32–1.20)	0.27 (0.13-0.57) **	0.43 (0.21-0.87) *	0.003
No	*n* (cases/controls)	182/130	104/129	64/130	86/130	
	OR (95% *CI*) ^1^	1.00	0.52 (0.36–0.76) **	0.33 (0.22–0.50) ***	0.40 (0.27–0.59) ***	<0.001
*P*_–interaction_						0.912
Tea drinking						
Yes	*n* (cases/controls)	148/100	79/99	56/99	70/100	
	OR (95% CI) ^1^	1.00	0.58 (0.37–0.89) *	0.40 (0.25–0.64) ***	0.43 (0.27–0.67) ***	<0.001
No	*n* (cases/controls)	113/62	75/61	46/61	57/62	
	OR (95% CI) ^1^	1.00	0.58 (0.35–0.95) *	0.30 (0.17–0.53) ***	0.42 (0.25–0.71) **	<0.001
*P*_–interaction_						0.366

*** *p* < 0.001; ** *p* < 0.01; * *p* < 0.05; ^+^ *p* < 0.1; ^1^ adjusted for sex, age, BMI, education level, income level, smoking, alcohol drinking, tea drinking, and physical activity.

## Supplementary Materials

The following are available online at http://www.mdpi.com/2072-6643/8/10/624/s1, Table S1: Odds ratios (OR) and 95% confidence intervals (CI) of primary liver cancer according to quartiles of carotene-rich and retinol-rich food intake, Table S2: Food contents of RE and the amount of foods providing vitamin A of 1000 μg RE.

## Abbreviations

PLC	primary liver cancer
HBV	hepatitis B virus
HCV	hepatitis C virus
VA	Vitamin A
HCC	hepatocellular carcinoma
ATBC	the Alpha-Tocopherol, Beta-Carotene Cancer Prevention Study
CARET	Beta-Carotene and Retinol Efficacy Trial
GPS	generalized propensity score
NCCN	National Comprehensive Cancer Network
FFQ	food frequency questionnaire
OR	odds ratios
RE	retinol equivalent
SELECT	the Selenium and Vitamin E Cancer Prevention Trial
ROS	reactive oxygen species
RNS	reactive nitrogen species
8-OhdG	8-hydroxydeoxyguanosine
HSC	hepatic stellate cells
MET	metabolic equivalent
WHR	waist-to-hip ratio retinol
BMI	Body mass index
