# Supplementary material for: The Association between Dietary Vitamin A and Carotenes and the Risk of Primary Liver Cancer: A Case–Control Study
Source: Nutrients. 2016 Oct 11;8(10):624. doi: 10.3390/nu8100624 (PMC5084012; doi:10.3390/nu8100624)
Supplement: Supplementary file 1 [file nutrients-08-00624-s001.docx]

Supplementary Materials: The Association between Dietary Vitamin A and Carotenes and the Risk of Primary Liver Cancer: A Case–Control Study

Qiu-Ye Lan, Yao-Jun Zhang, Gong-Cheng Liao, Rui-Fen Zhou, Zhong-Guo Zhou, Yu-Ming Chen and Hui-Lian Zhu

**Table S1.** Odds ratios (OR) and 95% confidence intervals (CI) of primary liver cancer according to quartiles of carotene-rich and retinol-rich food intake.

|  | **Amount (g/Day)** | ***n* (Cases/Controls)** | **Univariate Model OR (95% CI)** | **Model 1 OR (95% CI)** |
| --- | --- | --- | --- | --- |
| Quartile of leafy green vegetables | | | | |
| Q1 | ≤115.43 | 274/161 | ref | ref |
| Q2 | 115.43–182.24 | 162/161 | 0.59 (0.44–0.79) *** | 0.59 (0.43–0.81) ** |
| Q3 | 182.24–276.48 | 110/161 | 0.40 (0.29–0.55) *** | 0.41 (0.29–0.57) *** |
| Q4 | >276.48 | 98/161 | 0.36 (0.26–0.49) *** | 0.38 (0.27–0.53) *** |
| *P*-trend |  |  | <0.001 | <0.001 |
| Quartile of carrots | | | | |
| Q1 | ≤3.95 | 225/161 | ref | ref |
| Q2 | 3.95–9.78 | 115/161 | 0.51 (0.37–0.70) *** | 0.52 (0.37–0.73) *** |
| Q3 | 9.78–21.86 | 150/161 | 0.67 (0.49–0.90) ** | 0.68 (0.49–0.93) * |
| Q4 | >21.86 | 154/161 | 0.68 (0.51–0.92) * | 0.67 (0.48–0.93) * |
| *P*-trend |  |  | 0.032 | 0.041 |
| Quartile of eggs | | | | |
| Q1 | ≤12.31 | 225/161 | ref | ref |
| Q2 | 12.31–25.37 | 164/161 | 0.73 (0.54–0.98) * | 0.77 (0.56–1.07) |
| Q3 | 25.37–43.52 | 122/161 | 0.54 (0.40–0.74) *** | 0.55 (0.39–0.77) ** |
| Q4 | >43.52 | 133/161 | 0.59 (0.44–0.80) ** | 0.55 (0.40–0.78) ** |
| *P*-trend |  |  | <0.001 | <0.001 |
| Quartile of animal offal | | | | |
| Q1 | ≤0 | 412/237 | ref | ref |
| Q2 | 0–0.56 | 17/85 | 0.11 (0.06–0.19) *** | 0.13 (0.07–0.22) *** |
| Q3 | 0.56-2.63 | 74/161 | 0.26 (0.19–0.36) *** | 0.25 (0.18–0.35) *** |
| Q4 | >2.63 | 141/161 | 0.50 (0.38–0.66) *** | 0.44 (0.33–0.60) *** |
| *P*-trend |  |  | <0.001 | <0.001 |

*** *p* < 0.001; ** *p* < 0.01; * *p* < 0.05; Model 1: adjusted for sex, age, BMI, education level, income level, smoking, alcohol drinking, tea drinking, and physical activity.

**Table S2.** Food contents of RE and the amount of foods providing vitamin A of 1000 μg RE.

| **Vegetables** | **Contents μg RE/100 g** | **Food g/1000 μg RE** | **Fruits** | **Contents μg RE/100 g** | **Food g/1000 μg RE** |
| --- | --- | --- | --- | --- | --- |
| Spinach | 487 | 205 | Orange | 148 | 676 |
| Spring cabbage | 757 | 174 | Mango | 150 | 667 |
| Broccoli | 1202 | 83 | Papaya | 145 | 690 |
| Carrot | 688 | 145 | Cantaloupe | 153 | 653 |
| Watercress | 1592 | 63 | Persimmon | 73 | 1370 |
| Boxthorn leaf | 592 | 169 | Watermelon | 75 | 1333 |
| Pea seedlings | 445 | 224 |  |  |  |
| Cayenne pepper | 232 | 430 |  |  |  |
| Fragrant-flowered garlic | 235 | 426 |  |  |  |
| Beet leaves | 610 | 164 |  |  |  |
| Citronella | 500 | 200 |  |  |  |
